# Genomic variants associated with type 2 diabetes mellitus among Filipinos

**DOI:** 10.1371/journal.pone.0312291

**Published:** 2024-11-19

**Authors:** Eva Maria C. Cutiongco-de la Paz, Jose B. Nevado Jr., Elizabeth T. Paz-Pacheco, Gabriel V. Jasul Jr., Aimee Yvonne Criselle L. Aman, Mark David G. Francisco

**Affiliations:** 1 Institute of Human Genetics, National Institutes of Health, University of the Philippines Manila, Manila, Philippines; 2 Philippine Genome Center, University of the Philippines, Manila, Philippines; 3 Department of Medicine, Division of Endocrinology, Philippine General Hospital, University of the Philippines Manila, Manila, Philippines; University of North Carolina at Chapel Hill, UNITED STATES OF AMERICA

## Abstract

Type 2 diabetes mellitus leads to debilitating complications that affect the quality of life of many Filipinos. Genetic variability contributes to 30% to 70% of T2DM risk. Determining genomic variants related to type 2 diabetes mellitus susceptibility can lead to early detection to prevent complications. However, interethnic variability in risk and genetic susceptibility exists. This study aimed to identify variants associated with type 2 diabetes mellitus among Filipinos using a case-control design frequency matched for age and sex. A comparison was made between 66 unrelated Filipino adults with type 2 diabetes mellitus and 121 without. Genotyping was done using a candidate gene approach on genetic variants of type 2 diabetes mellitus and its complications involving allelic association and genotypic association studies with correction for multiple testing. Nine (9) significant variants, mostly involved in glucose and energy metabolism, associated with type 2 diabetes mellitus in Filipinos were found. Notably, a *CDKAL1* variant (rs7766070) confers the highest level of risk while rs7119 (*HMG20A*) and rs708272 (*CETP*) have high risk allele frequencies in this population at 0.77 and 0.66, respectively, making them potentially good markers for type 2 diabetes mellitus screening. The data generated can be valuable in developing genetic risk prediction models for type 2 diabetes mellitus to diagnose and prevent the condition among Filipinos.

## Introduction

Type 2 diabetes mellitus is an important significant public health concern. The International Diabetes Federation Diabetes Atlas reported a 7.1% prevalence of type 2 diabetes mellitus in the Philippines. Mortality due to type 2 diabetes mellitus was recorded at 30,713 deaths in 2023, 39,193 deaths in 2022, and nearly 56,000 deaths in 2021. From 2021 to 2023, statistics of death due to diabetes mellitus accounts for about 6.3–6.5% share of the total all-cause mortality [1–3].

Hyperglycemia and chronic inflammation in type 2 diabetes mellitus may cause various organ dysfunction and systemic vasculopathy. Such chronic injuries affect vital organs depending on their predominant manifestation. This contributes proportionally higher mortality in lower-middle-income countries such as the Philippines [4]. Despite significant life-threatening complications, type 2 diabetes mellitus is still considered preventable and modifiable. By modification of diet and lifestyle, diabetes and its complications can be prevented, delayed, and/or alleviated. For most people, motivation for behavioral modification can be increased substantially if they are informed of their risk beforehand. An assessment that can identify susceptible individuals even before the onset of type 2 diabetes mellitus is critical.

Recent studies established the role of genetics in the development of type 2 diabetes mellitus across population groups with reported heritability between 30–70% [5]. Studies among different populations showcase interethnic variability in type 2 diabetes mellitus genetic susceptibility and risk for complications. However, there is a scarcity of data among Filipinos. As the validation of variants associated with one population seems suboptimal if applied to another population, designing a specific set of genetic variants for a particular population poses a challenge.

The study aimed to identify variants associated with type 2 diabetes mellitus among the Filipino population. Data generated can be valuable for developing genetic risk prediction models relevant to preventing and diagnosing the disease among Filipinos.

## Materials and methods

The study was implemented from March 2014 to December 2018, following the approval of the University of the Philippines Manila Review Ethics Board (UPMREB-2012-0185-NIH). Participants were enrolled from the Philippine General Hospital, government hospitals, local health centers, and private clinics within and outside Metro Manila. All subjects were given written informed consent forms to sign for inclusion and storage of biological samples for future use before they participated in the study.

### Study design

This case-control study compared participants with type 2 diabetes mellitus and controls to determine genetic variants associated with type 2 diabetes mellitus. To control for factors that may be affected by significant After screening using the inclusion and exclusion criteria (Supplemental Material 1), a total of 201 unrelated participants were enrolled.

### Clinical data collection

All participants had been tested for blood lipid profile, serum creatinine, AST, ALT, alkaline phosphatase, and C-peptide levels. Standard laboratory tests were performed at the Medical Research Laboratory, Philippine General Hospital. Ancillary procedures such as a 12-lead electrocardiogram, fundoscopy, and ankle-brachial index were also done. A urine sample was also collected for the albumin-creatinine ratio.

To measure the participant’s fasting blood glucose, glucose oxidase method was used. While for HbA1C, it was measured using high performance liquid chromatography (HPLC). Both HDL and LDL levels were measured using a direct enzymatic method while for cholesterol and triglyceride, CHOD-PAP cholinesterase and GPO-PAP enzymatic were used respectively (Table 1).

**Table 1:** Clinical profile of participants in the study.

Clinical Features	With T2DM (n = 66)	Without T2DM (n = 121)	p-value
Age (mean years ± SD)	55.18 (10.81)	55.69 (11.67)	*Ns*
Sex (% males)	33.85	32.79	*Ns*
Hypertension (%)	89.23	29.51	< 0.001
Obese (%)*	64.63	47.96	< 0.001
Anthropometrics			
BMI (mean kg/m^2^ ± SD)	25.33 (4.72)	27.04 (4.91)	0.027
Waist-hip ratio (mean ± SD)	0.957 (0.062)	0.922 (0.061)	< 0.001
Laboratory tests			
Fasting blood glucose (mg/dL± SD)	155.25 (83.82)	81.73 (9.53)	< 0.001
HBA1c, (% ± SD)	8.19 (1.92)	5.57 (0.42)	< 0.001
LDL-c (mean mg/dL ± SD)	120.22 (47.08)	138.81 (39.54)	< 0.001
HDL-c (mean mg/dL ± SD)	47.54 (13.53)	52.29(13.71)	0.024
Triglycerides (mean mg/dL ± SD)	150.60(102.26)	121.50 (90.81)	0.047

BMI>25; Abbrev: T2DM, Type 2 diabetes mellitus; FBS, fasting blood sugar; HbA1c, hemoglobin A1c; BMI, body mass index; LDL-c, low-density lipoprotein cholesterol; HDL-c, high-density lipoprotein cholesterol; SD, standard deviation; ns, not significant.

*The p-value is significant at p < 0.05.

### DNA extraction and quantification

Blood samples were collected and stored in EDTA tubes at 4°C. DNA was extracted using the QiaAmp DNA Blood Mini Kit following the spin protocol specified in the manufacturer’s instruction manual. The eluted DNA was quantified using NanoDrop 2000 spectrophotometer (Thermo Fisher Scientific) at 260 nm and stored at 20°C until microarray genotyping. Only samples with A260/280 between 1.8 to 2.0 were considered viable for further processing.

### Design of customized microarray chips

A customized beadchip was designed using candidate SNPs from both coding and non-coding regions, including intergenic and intronic SNPs, which have shown statistical evidence of association with type 2 diabetes mellitus,its complications, treatment, diagnosis, and other related conditions (S1 and S2 Figs). Variants were selected from curated genome repositories and patent databases such as PharmGKB database, National Human Genome Research Institute (NHGRI), GWAS catalog, PubMed, Patentscope, and Espacenet where risk and protective odds rations were provided (S1 Table). Variants with crude odds ratios (ORs) of > 2.0 or < 0.5 and p-values <10^−5^ in GWAS were preferred, while other SNPs with less established or smaller ORs were also included to assess the frequency of these alleles among Filipinos. The variants were scored to determine the suitability to discriminate and estimate specificity.

### Genotyping

Customized genotyping of candidate SNPs was performed using DNA microarray technology following the GoldenGate Genotyping and Illumina Infinium iSelect assay protocols specified in their respective manufacturer’s manual. Screening for SNPs among genes clinically associated with type 2 diabetes mellitus and its complications was done by imaging beadchips on the HiScan system and utilizing the GenomeStudio v2.0 software.

As two different microarray protocols were performed, GenomeStudio v2.0 and Stata/MP v14.1 were used to consolidate a merged list of relevant SNPs. This strategy was done as per manufacturer’s manual of Illumina Infinium iSelect, using updated SNP rs IDs. After evaluation, 351 candidate SNPs were included for further selection.

### Quality control

GenomeStudio v2.0 was used to evaluate sample data quality and filter participants and SNPs with incomplete data. Only genotyping data with a call frequency ≥ 95% from samples with a call rate ≥ 95% were included in the study (Illumina, 2008).

To evaluate the completeness of participant and SNP data, gPLINK v2.05.10 was used. Participants with an individual missingness rate of more than 10% were excluded from further analysis. Further SNPs were excluded according to the following conditions: minor allele frequency less than 1%, genotype missingness rate more than 10%, and significant Hardy-Weinberg disequilibrium among controls p<0.001. After quality control and clean-up, the remaining variants underwent allelic and genotypic association analysis.

### Data analyses methods

#### Profiling clinical and baseline data

Stata 14.1 (College Station, TX: StataCorp LP) was used to compare demographic and clinical data of case and control groups with p-value set at p≤0.05. Two-sample Student’s t-tests were done for continuous data and chi-square test for categorical data.

#### Allelic association

Determination of risk allele and risk allele frequency was performed through allelic association tests on gPLINK 2.050. Statistics used Fisher-Irwin exact tests with correction for multiple testing was done via Holm-Bonferroni adjustments, when possible.

The crude ORs infer the impact of an allele on phenotypic outcome. An OR > 1.0 implied a susceptibility (risk), whereas an OR < 1.0 implied protection. If the OR was > 1.0, the minor allele was reported as the risk allele, and the minor allele frequency (MAF) was reported as the risk allele frequency (RAF). If the OR was < 1.0, the major allele was reported as the risk allele. The reciprocal of the OR (1 ÷ OR, ROR) would be the risk ROR, and the percent difference of the MAF was reported as the RAF.

#### Genotypic association

For genotypes, allelic combinations were assessed using prototypical models as follows: an additive model, when the presence of more copies of the risk allele confers a higher risk of the complication or associated disease; a recessive model, when the presence of two risk alleles is required for the trait/phenotype to be expressed; and a dominant model, when the presence of only one risk allele is needed for the trait/phenotype to be expressed. These models were inferred based on the distribution of the case and control genotypes among participants. Fisher-Irwin exact tests of association determine the best possible mode of genetic effect at a nominally significant p-value < 0.05. Correction for multiple testing was via Holm-Bonferroni adjustments.

#### Multiple logistic regression

The comorbidities and clinical parameters with p-value < 0.2 (Table 1) and significant SNPs resulting from the genotypic and allelic association analysis were used as variables to generate the full model. To generate the final prediction model from the full model, multiple logistic regression analysis was performed in R v4.4.1 using the package MASS. The variables with p-value set at ≤ 0.05 were retained for the final/parsimonious model. The regression analysis excluded data that are considered complications or surrogates of type 2 diabetes mellitus.

## Results

**Fig 1:**
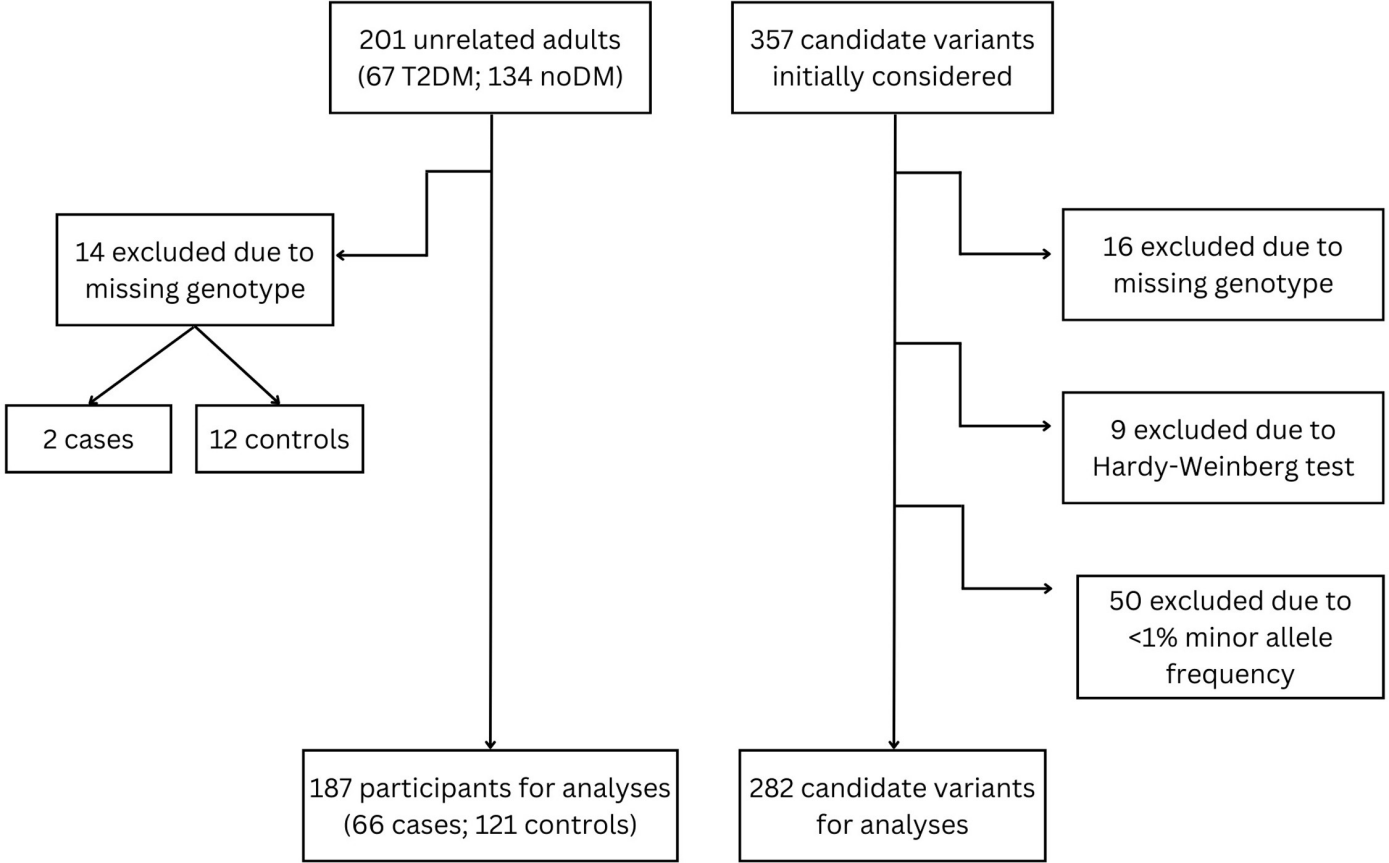
Schema for data cleanup of the samples and variants in the study.

After recruitment and data quality control, 187 out of the 201 samples were included, of which 66 were cases with type 2 diabetes mellitus and 121 controls. Of the initial 357 candidate variants, only 282 variants were further investigated after data clean-up (Fig 1).

Type 2 diabetes mellitus cases had an average of 16 years from the time of diagnosis, with a mean FBS level of 155.25 mg/dL and a mean HbA1c of 8.19%. The cases also had a significantly higher prevalence of hypertension and obesity than controls (p<0.001). LDL-C levels were found to be lower among cases than controls. This may be due to the use of LDL-targeted anti-hyperlipidemic medications among diabetic participants being part of standard care, with most having taken statins (51.52%) compared to controls (5%; p < 0.001).

Nine variants were found to be significantly associated with type 2 diabetes mellitus in the study after multiple testing-corrected statistics (Holm-Bonferroni adjusted p-value) (Table 2). Most variants are located outside known coding regions, many of which are likely regulatory. Variants rs699 in the *AGT* gene and rs429358 in the *APOE* gene are located in the coding region and are missense variants, reported to have a loss-of-function effect on the genes. Interestingly, the risk allele is the major allele in most of the variants except for rs7766070 and rs12150053, indicating that the nine variants are generally expected in the population who have type 2 diabetes mellitus.

**Table 2:** Allelic characterization of variants significantly associated with T2DM in the study.

Variants	Implicated Genes (chrom loc)	Function	Risk allele (% cases vs. control))	Allelic OR (95% CI)	Adjusted p-value*
rs7766070	*CDKAL1* (6p22.3)	Intron / regulatory region variant	A (37.9 vs. 5.9)	9.67 (5.08–18.42)	1.55e-11
rs391300	*SRR* (17p13.3)	Intron / upstream gene variant	G (99.1 vs. 66.0)	71.89 (9.87-inf)	5.64e-11
rs708272	*CETP* (16q13)	Intron / regulatory region variant	C (100 vs. 66.1)	128.17 (7.87-inf)	9.73e-11
rs2383208	*CDKN2B-AS1* (9p21.3)	Downstream gene variant	G (73.5 vs. 36.8)	4.76 (2.99–7.60)	5.09e-09
rs7119	*HMG20A* (15q24.3)	3’ UTR / non-coding transcript exon / downstream gene / regulatory region variant	G (100 vs. 79.6)	70.41 (4.30-inf)	4.54e-06
rs12150053	*SERPINF1* (17p13.3)	Upstream gene variant	C (27.3 vs. 6.3)	5.58 (2.92–10.66)	6.08e-06
rs659366	*UCP2* (11q13.4)	Upstream gene / non-coding transcript exon / regulatory region / TF binding site variant	C (100 vs. 78.8)	69.88 (4.27-inf)	7.77e-06
rs699	*AGT* (1q42.2)	Missense variant M (ATG) >T (ACG)	C (100 vs. 86.7)	41.43 (2.51-inf)	2.51e-03
rs429358	*APOE* (19q13.32)	Missense variant C (TGC) > R (CGC)	C (8.3 vs. 0.4)	21.91 (2.80–171.7)	6.38 e-03

*The p-value is significant at p<0.05 after the Holm-Bonferroni adjustment.

Abbr. CDKAL1, CDK5 regulatory subunit associated protein 1 like 1; SRR, serine racemase; CETP, cholesteryl ester transfer protein; CDKN2B-AS1, cyclin dependent kinase inhibitor 2B antisense RNA; HMG20A, high mobility group 20A; SERPINF1, serpin family F member 1; UCP2, uncoupling protein 2; AGT, angiotensinogen; APOE, apolipoprotein E.

The SNPs with the highest allelic ORs were rs708272 in the *CETP* gene (128.17, 95%CI: 7.87–2088.33), rs391300 in the *SRR* gene (71.89, 95%CI: 9.87–524.11), rs7119 in the *HMG20A* gene (70.41. 95%CI: 4.30–1151.85), rs659366 near *UCP2* (69.88; 95%CI: 4.27–1142.97), and rs699 in the *AGT* gene (41.43, 95%CI: 251–682.86). Two genes were very proximal to each other located on chromosome 17p13.3 ‐ rs12150053 near *SERPINF1* and rs659366 near *UCP2*, suggesting a possible hotspot related to susceptibility to type 2 diabetes mellitus in the region.

Genotypic analyses showed that most of the derived variants fit the dominant-recessive model (Table 3). This is probably due to the study sample size that permits the detection of models with less sensitivity to others, such as the additive or the multiplicative model, which may require larger sizes. Five dominant variants, rs7119-A, rs708272-T, rs391300-A, rs659366-T and rs699-T have protective effects, while rs7766070-A, rs12150053-C, rs2383208-G, and rs429358-C have risk effects. The risk allele frequencies presented in Table 4, are placed side by side with the frequencies of the alleles from different populations included in the 1000 Genomes Project for comparison. Only rs7766070, rs12150053, and rs429358 were rare at <10% minor allele frequency. The common alleles (risk allele frequency >50%) also appear to be expected in other populations.

**Table 3:** Genotypic features of variants significantly associated with T2DM in the study.

Variants	Genotypes (risk vs. non-risk)	Reference genotype frequency (%cases vs %control)	Genotypic OR* (95% CI)	p-value**
rs7766070	AA and AC vs. CC (A- dominant for DM)	66.7 vs. 10.2 (AA and AC)	17.67 (8.05–38.78)	3.89e-13
rs391300	GG vs. AA and AG (A- dominant for noDM)	1.8 vs 58.7 (AA and AG)	0.013 (1.69e-03-0.094)	1.82e-11
rs708272	CC vs. CT and TT (T- dominant for noDM)	0 vs. 52.1 (CT and TT)	7.8e-03 (e-inf -0.13)	4.73e-11
rs2383208	GG and AG vs. AA (G- dominant for DM)	100 vs 54.5 (GG and AG)	4.77 (2.98 ‐ inf)	3.29e-09
rs7119	GG vs. AG and AA dominant for noDM)	0 vs 39.5 (AG and AA)	0.14 (e-inf ‐ 0.24)	2.02e-06
rs12150053	CC and CT vs. TT (C- dominant for DM)	47.0 vs. 10.9 (CC and CT)	7.22 (3.41–15.32)	5.90e-06
rs659366	CC vs. CT and TT (T- dominant for noDM)	0 vs. 39.2 (CT and TT)	0.014 (e-inf -0.24)	3.00e-06
rs699	CC vs. TT and CT (T- dominant for noDM)	0 vs. 24.2 (TT and CT)	0.023 (e-inf -0.40)	3.92e-03
rs429358	CC and CT vs. TT (C -dominant for DM)	12.1 vs. 0.8 (CC and CT)	16.55 (2.02–135.5)	9.30e-03

*Computed using logistic regression.

**The p-value is significant at p<0.05 after Holm-Bonferroni adjustment; computed using Fisher exact t-test.

**Table 4:** Risk alleles of T2DM-associated variants and their frequencies compared with other populations from the 1000 Genomes Project.

Variants	Risk allele			Risk allele frequencies				
		Study*	Global	AFR	AMR	EAS	EUR	SAS
rs7766070	A	0.04	0.27	0.2	0.24	0.39	0.28	0.27
rs391300	G	0.64	0.55	0.46	0.65	0.68	0.61	0.43
rs708272	C	0.66	0.62	0.75	0.54	0.63	0.58	0.55
rs2383208	G	0.37	0.21	0.17	0.14	0.42	0.17	0.13
rs7119	G	0.77	0.66	0.52	0.62	0.85	0.59	0.74
rs12150053	C	0.06	0.21	0.03	0.22	0.24	0.39	0.22
rs659366	C	0.77	0.59	0.55	0.58	0.58	0.64	0.63
rs699	C	0.87	0.71	0.90	0.64	0.85	0.41	0.64
rs429358	C	0.03	0.15	0.27	0.10	0.09	0.16	0.09

Abbrev: AFR, African; AMR, admixed American; EAS, East Asian; EUR, European; SAS, South Asian.

*Presented are the risk allele frequencies among the control group of this study, compared with the findings from the 1000 Genomes Project.

Table 5 shows the significant variables affecting the prediction model generated in the study. The variable with the lowest p-value is hypertension, while the SNP with the lowest p-value of 0.00826 is rs391300 of the *SRR* gene. The odds ratio (OR) of the mentioned SNP is less than 1, indicating possible protective effects of the *rs391300-A* variant. On the contrary rs7766070 of the *CDKAL1* gene has an OR greater than 1 which may indicate a risk effect on the population for type 2 diabetes mellitus. This observation is also true for hypertension and obesity having an OR of 66.54 and 16.53, respectively.

**Table 5:** Parsed model for the multivariate analysis in the study.

Variable	Genotype	Adjusted OR (95% CI*)	p-value
Hypertension	-	66.54 (7.496, 1254.295)	0.000985
Obesity	-	16.43 (1.566, 342.176)	0.036525
LDL-C	-	0.04 (0.003, 0.344)	0.009024
rs7766070	AA vs CC	39.53 (5.213, 643.845)	0.00834
rs391300	AA vs GG	0.0005 (4.44ER-07, 0.097)	0.00826
rs659366	CT vs CC	0.004 (1.16E-05, 0.921)	0.03837

*OR–odds ratio, CI–confidence interval.

## Discussion

Genetic studies are essential in understanding the pathogenesis of diseases and discovering clinical applications. Genetic susceptibility studies have been done in different populations to predict the risk of Type 2 diabetes mellitus as well as its debilitating complication, and to develop strategies for prevention. Considering that inter-ethnic genetic variations exist, this study investigated Type 2 diabetes mellitus susceptibility variants in Filipinos using a candidate gene approach.

The study found nine variants associated with type 2 diabetes mellitus among Filipinos. Most variants are related to metabolic processes, including glucose metabolism. Briefly, descriptions of the variant, emphasizing possible metabolic roles and their reported associated genes, are presented below.

### rs7766070

This variant located in intron 5 of *CDKAL1* (Cyclin Dependent Kinase 5 regulatory subunit associated protein 1 like 1) encodes a protein member of the methylthiotransferase family. Because *CDKAL1* has similar protein domains to *CDKRAP1*, it may have the same adverse regulatory effects of *CDKRAP1* towards CDK5 via inhibition of CDK5 activator p35 [6]. A suggested mechanism of action would be through an action involving the neuroenteric and neuroinsular axes affecting the glucometabolic metabolism through the activity of beta cells and glucose-dependent insulinotropic peptide (GIP), which inhibits lipolysis and increases insulin response [7]. According to the GTEx portal (https://gtexportal.org), the expression ratio of *CDKAL1* with the AA and AC genotype is greater than with CC genotype possibly indicating upregulated expression when risk variant *rs766070-A* is present.

This study among Filipinos shows that study participants with AA and AC genotypes for *rs7766070* are more likely to have type 2 diabetes mellitus than those with the CC genotypes, which is consistent with previous findings among the Lebanese and in a multi-ethnic cohort [8]. Moreover, *CDKAL1* is also associated with gestational diabetes [9]. Interestingly, several polymorphic variants (*rs7756992*, *rs7754840*, and *rs10946398*) are also located in the non-coding region of the *CDKAL1* locus, which is strongly associated with an increased risk of developing diabetes among those with European ancestry [10]. These suggest that *CDKAL1* gene expression affects an individual’s susceptibility to Type 2 diabetes mellitus.

### rs391300

This variant is in the first intron of the gene serine racemase (*SRR*), an enzyme essential in serine biosynthesis. Its likely association with type 2 diabetes mellitus is the regulatory role of serine in the actions of N-methyl-D-aspartate (NMDA) receptors, which inhibit insulin secretion by pancreatic beta cells [11]. Increased expression of *SRR* has been observed in type 2 diabetes mellitus similar to results in the GTEx portal database showing that the expression of rs391300 where T is the risk allele (TT and TC genotypes) has an increased expression ratio when compared to the CC genotype. From this, it is possible that gene expression is upregulated when mentioned genotype of variant is present [12]. *SRR* involves cellular memory, lipid and lipoprotein metabolism pathways, glycine, serine, and threonine metabolism, and biosynthesis [13]. Subsequent analyses suggest a role in lipid catabolism in adipocytes, insulin resistance, and amino acid metabolism. This study among Filipinos found similar findings to the study among Han Chinese where the G allele conferred risk of developing Type 2 diabetes mellitus [14]; however, this association is not seen among the Japanese.

### rs708272

This variant, also called Taq1B polymorphism, is located in the first intron of *CETP* (cholesteryl ester transfer protein gene), a carrier enzyme responsible for transporting cholesterol esters and triglycerides between VLDL, LDL, and HDL. Low levels of *CETP* promote HDL formation and reduce the risk for cardiovascular events [15].

In this study, the CC genotype more likely confers susceptibility to type 2 diabetes mellitus than those with the TC and TT genotypes. This finding is similar to previous studies which identified the G as the risk allele and A as the alternative [16], as the study used the complementary sequences corresponding to the risk allele B1B1 for the CC genotype. This polymorphism has been associated with metabolic syndrome [17] and type 2 diabetes mellitus risk among those with hyperlipidemia [18]. The variant is associated with low HDL among Koreans and risk for coronary atherosclerosis among Chinese [15,19]. Interestingly, Taq1B B1B1 polymorphism (CC genotype) is linked with low HDL-C with known cardiovascular risk factors including type 2 diabetes mellitus among Filipinos [20].

In addition, when comparing expression levels of *CETP* gene, the CC genotype appears to be downregulated as compared to the TC and TT genotypes having a greater normal expression ratio (https://gtexportal.org).

### rs2383208

This variant is found 11kb downstream from *CDKN2B-AS1* (cyclin-dependent kinase inhibitor 2B antisense RNA 1) in the *CDKN2B-CDKN2A* gene cluster on chromosome 9, a significant locus for genetic susceptibility to cardiovascular disease, type 2 diabetes mellitus, and cancer [21]. *CDKN2B-AS1* is a non-coding gene that produces a functional RNA that interacts with polycomb repressive complexes (PRCs) that lead to the epigenetic silencing of genes within the *CDKN2B-CDKN2A* gene cluster [22]; this *CDKN2B-AS1* variant may indirectly affect the silencing of type 2 diabetes mellitus-associated genes in the *CDKN2B-CDKN2A* cluster possibly resulting in the lower gene expression levels of *CDKN2B-AS1* when the risk variant rs2383208 with *GG* genotype is present [23].

Not much is known about the direct association of rs2383208 with type 2 diabetes mellitus. An increase in the expression of *ANRIL*, a regulatory aspect of *VEGF* expression, in diabetic blood samples was noted during hyperglycemia in the retina of mice models indicating a protective effect [24]. Other studies associate the variant with the risk of developing Type 2 diabetes mellitus vascular complications, including coronary artery disease (CAD) [25], retinopathy [26], and nephropathy [27].

*CDKN2B-AS1* has been implicated among several significant risk loci for type 2 diabetes mellitus in GWAS studies done among the Japanese, Indian, and Han Chinese where the AG and GG genotypes confer risk compared with the AA phenotype, differing from the previously reported risk allele A [14,28]. This study among Filipinos similarly produced a trend towards a more significant association of the G allele with CAD in Type 2 diabetes mellitus among the Han Chinese. These differences make evident interethnic variation for this particular variant.

### rs7119

This variant is in exon 11 of *HMG20A* (high mobility group 20 A), a gene responsible for regulating metabolism-secretion coupling genes and the functional maturity of beta cells. Its expression is decreased in the pancreatic beta cells of diabetes, with experimental depletion resulting in blunting of glucose-induced insulin response and promoting beta cell de-differentiation [29]. As the gene is a putative chromatin factor, it is speculated to be crucial in beta cell maturation and homeostasis. Additionally, according to the GTEx portal database, *HMG20A* expression is lower in variants with the CC genotype in contrast to the higher gene expression ratios of the TT and TC genotypes, with T as the risk allele.

A previous study showed that rs7119 is associated with type 2 diabetes mellitus among persons with Chinese, Malay, and Indian ancestry. Contrary to a study done by Sim et al. (2011), the T (vs. C) allele was identified as the risk allele whereas the present study among Filipinos showed G (vs. A) as the risk allele in the type 2 diabetes mellitus group. This discrepancy may have resulted from the difference in sample population as compared to the more homogenous Filipino population in the present study [30]. This would explain not only the difference in results, but also the more robust findings (OR of 1.12 vs 0.14 in the present study). Previous results among the Han Chinese did not show any association of this variant with type 2 diabetes mellitus, with one meta-analysis study also failing to demonstrate an association between rs7119 and type 2 diabetes mellitus risk in a Chinese population [31].

### rs12150053

This variant is a 2kb upstream of the serpin family F member 1 (*SERPINF1*) gene. It encodes a neurotrophic protein pigment epithelium-derived factor (*PEDF*) that induces neuronal differentiation and serves as a potent inhibitor of angiogenesis. GTEx Portal expression data associate the SNP with the higher gene expression levels of the SMYD4 gene with the CC genotype as compared to the lower expression levels with the TT and TC genotype. Superpathways related to *SERPINF1* are extensively involved in anti-angiogenesis, apoptosis, and cell signaling such as in Wnt signaling apoptosis/ docosahexaenoic acid (DHA) signaling, and PEDF signaling [32].

Current views suggest a protective effect of *PEDF* against diabetic complications through anti-oxidative and anti-angiogenetic mechanisms [33]. However, high levels may be detrimental to existing vasculopathies because of its association with diabetic retinopathy (DR) and high levels of PEDF related to renal failure progression in type 2 diabetes mellitus patients [33,34].

In the present study, the C allele in the rs12150053 variant is associated with an increased risk for type 2 diabetes mellitus among Filipinos. Similarly, a study among the Japanese showed a significant increase of the C allele frequency in the presence of DR [33]. The direct association of the variant to type 2 diabetes mellitus cannot be inferred due to the minimal information available at hand but the possibility of the variant as a confounder is considered due to its association with diabetic retinopathy, a common complication of type 2 diabetes mellitus. Thus, determination of the direct association of the variant to type 2 diabetes mellitus warrants further verification.

### rs659366

This variant is an upstream polymorphism of *UCP2* (uncoupling protein 2), which codes for a mitochondrial transporter protein present in pancreatic islet cells. *UCP2* uncouples ATP production in oxidative phosphorylation involved in glucose/energy metabolism and FOXA2 and FOXA3 transcription factor network in the insulin and insulin-like signaling pathways [35]. It is hypothesized that in type 2 diabetes mellitus pathogenesis, oxidative stress induces the expression of *UCP2*, resulting in reduced ATP synthesis in beta cells that results in slower insulin response to glucose levels [36]. Together with age-related gradual peripheral mitochondrial dysfunction, accumulated injury results in the gradual development of insulin resistance as well as a gradual loss of insulin responsiveness [37]. Moreover, an increased *UCP2* expression in white fat in adipocytes may provide a molecular link between type 2 diabetes mellitus and obesity [38]. GTEx portal associates the SNP with higher expression levels of the UCP2 with genotype TT in contrast with the lower expression with the TC and CC genotypes.

In this present study, the allele C was found to be the risk allele, similar to a study in Brazil which examined the association of this UCP2 variant with type 2 diabetes mellitus and diabetic retinopathy [39]. However, studies involving Iranians, South Asians, and Han Chinese did not find an association of type 2 diabetes mellitus with *UCP2* variants [36].

### rs699

This missense variant is located in the angiotensinogen (*AGT*) gene. The gene encodes for a potent vasoconstrictor and fluid and electrolyte regulator. The circulating molecule is central in the renin-angiotensin-aldosterone system (RAAS). Over-activation of RAAS interferes with insulin action in blood vessels, muscles, and fats resulting in insulin resistance leading to gradual system breakdown by inducing endothelial dysfunction and the development of vascular complications [40]. Moreover, one factor that promotes insulin resistance is chronic stress caused by long-term exposure to stress hormones and pathways, prominent among which are the renin-angiotensin-aldosterone pathways [41]. In the GTEx portal, an increased level of gene expression of *AGT* with the *GG* genotype is observed compared to the decreased levels of expression with the *AA* and *AG* genotypes, with G as the risk allele (https://gtexportal.org).

Vasculopathies due to dysfunctional regulation of the renin-angiotensin-aldosterone pathway have been linked to systemic vasculopathy in type 2 diabetes mellitus including nephropathy, coronary heart disease, cerebrovascular disease, peripheral arterial disease, and retinopathy [42]. In patients with kidney diseases associated with diabetic nephropathy and hypertension, rs699 gene polymorphisms are linked with end-stage renal disease development and progression [43]. The C risk allele in the present study among Filipinos corresponds to the missense mutation (M[ATG] > T[ACG] at amino acid position 235) in the *AGT* gene, the consensus risk variant for nephropathy [44]. Hence, the enrichment of the *AGT* variants in the type 2 diabetes mellitus group is likely possible due to its association with complications of type 2 diabetes mellitus.

Association studies of rs699 showed reduced risk of type 2 diabetes mellitus complications among Malays contrary to the result observed in the Tunisians and Pakistanis. The difference in results from studies of complex diseases such as diabetes may be caused by polygenic inheritance and gene-environment interaction distinction among different sets of populations [45].

### rs429358

This is a missense variant in the apolipoprotein E (*APOE*) gene, a major component of remnant circulating lipoproteins transporting lipids for hepatic clearance. In neuronal cells, *APOE* has isoforms that facilitate the peripheral distribution of lipids [46].

The variant rs429358 is most commonly associated with familial dysbetalipoproteinemia/type III hyperlipoproteinemia. It is commonly called the second loci for the epsilon alleles, essential in differentiating *ε2* and *ε4* alleles [47]. The *T* allele is a risk allele for high blood lipids while *C* is a risk allele for several neuronal-related diseases (e.g., Alzheimer’s disease and cerebrovascular diseases). The C to T variant substitution (Arg to Cys change) confers less binding affinity of lipids to peripheral APOE receptors [48]. The Arg158Cys (*ε2* allele) substitution produces a subtle conformational change influencing the binding of the LDL receptor resulting to a poor clearance of TG-rich lipoproteins from the plasma while the substitution Cys112Arg (*ε4* allele) induces a conformational change influencing the binding of lipid profiles [49]. In effect, *APOE* polymorphisms may be involved in the regulation of overall metabolic abnormalities (e.g. impaired glucose metabolism, dyslipidemia, and type 2 diabetes mellitus). In the GTEx portal, gene expression levels of the *APOE* gene is higher with the CC and TC genotypes than with the TT genotype, possibly indicating that the presence of the risk allele increases the expression levels of gene.

High cholesterol levels may lead to chronic insulin elevation with the gradual development of resistance in the peripheral tissue. In Egyptians, the *ε4* allele was associated with type 2 diabetes mellitus and CVD risk among diabetics, although the *T* allele is associated with higher total cholesterol, excluding HDL [50]. A similar observation was noted among Saudi Arabians where the ε4/ε4 variant is a significant risk for type 2 diabetes mellitus [51]. The relationship with diabetes has been observed in a few studies and in all instances, the *C* allele is the risk allele similar to the findings in the present study among Filipinos.

### Multiple logistic regression

After multiple logistic regression, the significant variables that remain are the hypertension, LDL-C, obesity, and variants rs7766070, rs391300, and rs659366. The odds ratio of each variable indicates its multiplicative effect on the risk of type 2 diabetes mellitus. An odds ratio of 66.54 implies that there is approximately 67 times more risk of type 2 diabetes mellitus in patients that have hypertension compared to those than do not have this condition. On the contrary, an odds ratio of 0.0005 for patients with allele A of variant rs391300 implies that there is a 99.95% reduction in the odds of having type 2 diabetes mellitus compared to a patient that has the allele G of the same variant.

Instead of using the full model, it is preferred to use the parsimonious model wherein only variables that have a p-value of less than 0.05 are included in order to eliminate factors that may affect the power and efficiency of the predictive model.

Upon analysis of the parsed model, the components that remained after the multiple regression analysis are components of the metabolic syndrome (obesity, hypertension, and LDL-C levels). Interestingly, there may be enough independence and interdependence among the variables that could be used to profile people that are pre-disposed to risk of high LDL-C levels, obesity, and hypertension. This raises the question of whether these conditions are associated with one another and what possible associations these three factors have related to the risk or susceptibility to type 2 diabetes mellitus in addition to the presence of risk variants.

### Implications of the study

The identification of variants which can be used as markers for screening predisposed/at-risk individuals for type 2 diabetes mellitus can help in its prevention. The high-risk allele frequencies (>50%) of variants already identified across several ethnic populations indicate that the derived variants are likely helpful in screening at-risk individuals for type 2 diabetes mellitus. The generation of the prediction model using the identified variants may aid to achieve the goal of decreasing the incidence of type 2 diabetes mellitus. Moreover, encouraging at-risk individuals to adopt changes in lifestyle and eating behaviors that promote better health can also help reduce the risk of type 2 diabetes mellitus and its associated complications [52].

The study used a candidate gene approach which may have missed variants that could be unique to Filipinos. In addition, the findings need to be validated to address veracity and variations in this population and other population groups. Future steps also involve higher throughput technologies that can discover novel variants in this population to have a more comprehensive picture and to help identify other markers that can help in mechanistic interpolation through pathway analyses. It is also recommended to look into differences of susceptibility between men and women since some studies have shown that men are twice more likely to get type 2 diabetes mellitus than women.

Lastly, although genetic variants may be causal in nature, inference of association regarding causality should be taken cautiously since associated variants may be linked with causal regions but not causal by themselves. Therefore, a significant challenge is to prove these variants’ role in the pathogenesis of type 2 diabetes mellitus.

## Supporting information

S1 Fig (TIF)

S2 Fig (TIF)

S1 Data DM2 genotype generated data. (XLSX)

S1 Table SNP list from curated genome repositories and patent databases. (DOCX)

S1 File Supplemental Material 1 ‐ inclusion and exclusion criteria. (DOCX)
