# Supplementary figures and images for: Genomic variants associated with type 2 diabetes mellitus among Filipinos
Source: PLoS One. 2024 Nov 19;19(11):e0312291. doi: 10.1371/journal.pone.0312291 (PMC11575783; doi:10.1371/journal.pone.0312291)

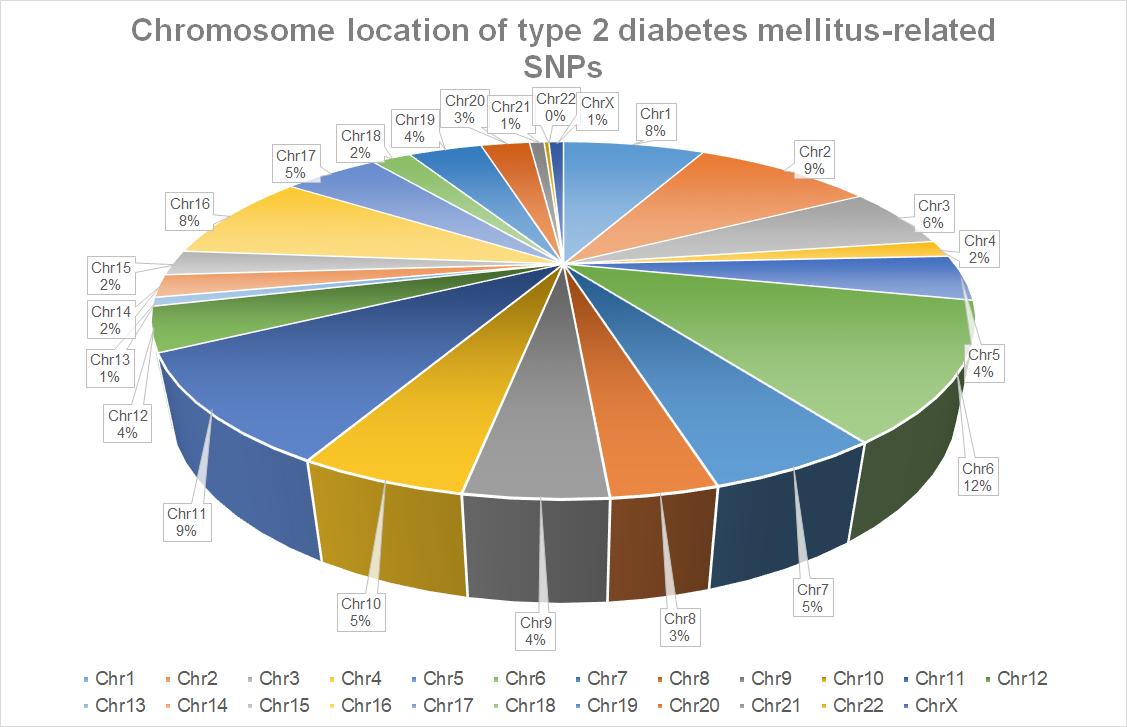

Supplement: S1 Fig — (TIF) [file pone.0312291.s001.tif]

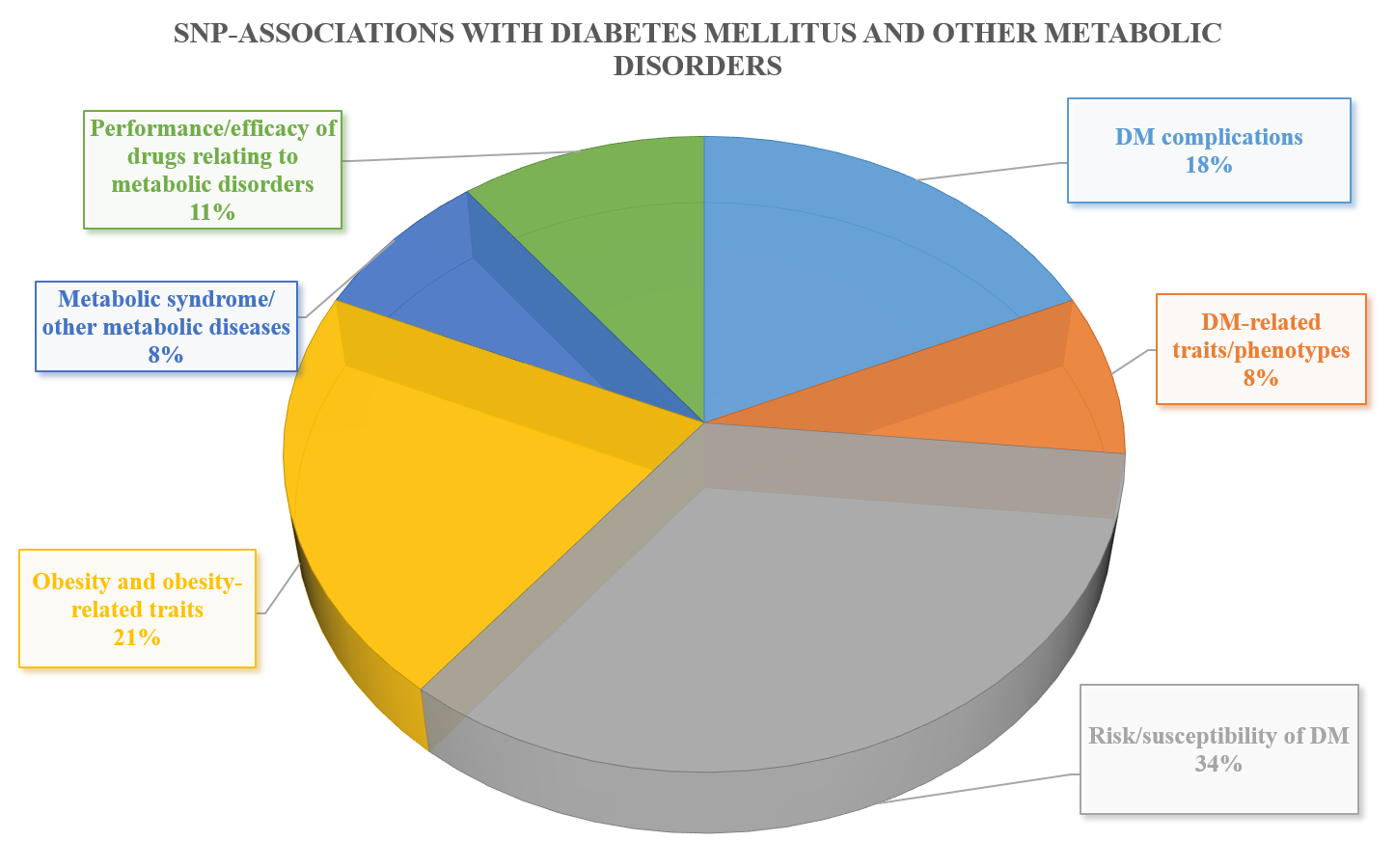

Supplement: S2 Fig — (TIF) [file pone.0312291.s002.tif]
