# Supplementary material for: Genomic variants associated with type 2 diabetes mellitus among Filipinos
Source: PLoS One. 2024 Nov 19;19(11):e0312291. doi: 10.1371/journal.pone.0312291 (PMC11575783; doi:10.1371/journal.pone.0312291)
Supplement: S1 Table — (DOCX) [file pone.0312291.s004.docx]

| Supplementary Table 1. SNP list from curated genome repositories and patent databases | | | | |
| --- | --- | --- | --- | --- |
| Chromosome | SNP ID | Genetic Position (Address/Centimorgans) | Physical Position (Number of Base-Pairs) | SNP |
| 7 | rs1002630 | 4931 | 29428070 | [T/C] |
| 14 | rs10151259 | 753 | 21790040 | [T/G] |
| 2 | rs10192566 | 23 | 11890428 | [G/C] |
| 19 | rs10401969 | 234 | 19407718 | [A/G] |
| 5 | rs1042713 | 2632 | 148206440 | [T/C] |
| 6 | rs1044498 | 5790 | 132172368 | [T/G] |
| 5 | rs10447248 | 5009 | 107915736 | [A/G] |
| 6 | rs10455872 | 2216 | 161010118 | [A/G] |
| 12 | rs1048466 | 1173 | 551550 | [T/C] |
| 6 | rs1048886 | 3484 | 71289189 | [T/C] |
| 14 | rs1048990 | 1227 | 35761675 | [C/G] |
| 1 | rs10494366 | 2485 | 162085685 | [A/C] |
| 2 | rs10497721 | 3980 | 192914362 | [A/C] |
| 10 | rs10508503 | 2983 | 16299951 | [A/G] |
| 10 | rs1057910 | 6021 | 96741053 | [T/G] |
| 20 | rs1059217 | 5221 | 45364649 | [A/G] |
| 10 | rs10741243 | 3030 | 132947962 | [G/C] |
| 9 | rs10757274 | 2901 | 22096055 | [A/G] |
| 11 | rs10767664 | 1746 | 27725986 | [A/T] |
| 15 | rs1077835 | 2081 | 58723426 | [A/G] |
| 11 | rs10790162 | 162 | 116639104 | [A/G] |
| 9 | rs10811661 | 585 | 22134094 | [T/C] |
| 9 | rs10818854 | 2122 | 126446778 | [T/C] |
| 11 | rs10830962 | 3704 | 92698427 | [C/G] |
| 11 | rs10830963 | 5052 | 92708710 | [C/G] |
| 11 | rs10891096 | 2934 | 110262743 | [T/G] |
| 3 | rs10937273 | 2158 | 186549695 | [A/G] |
| 12 | rs11066015 | 1886 | 112168009 | [A/G] |
| 12 | rs11066453 | 1808 | 113365621 | [A/G] |
| 12 | rs11109072 | 1954 | 97901270 | [T/G] |
| 10 | rs1111875 | 614 | 94462882 | [T/C] |
| 2 | rs11127485 | 4107 | 632028 | [T/C] |
| 3 | rs11128347 | 1931 | 73619561 | [C/G] |
| 1 | rs11208659 | 718 | 65979280 | [T/C] |
| 11 | rs11212617 | 1990 | 108283161 | [A/C] |
| 16 | rs1121980 | 442 | 53809247 | [T/C] |
| 16 | rs1132896 | 732 | 55519535 | [C/G] |
| 1 | rs1136410 | 1375 | 226555302 | [T/C] |
| 1 | rs1137101 | 4969 | 66058513 | [T/C] |
| 18 | rs11545881 | 1587 | 12274104 | [T/G] |
| 1 | rs11591147 | 2892 | 55505647 | [A/C] |
| 16 | rs11643718 | 2794 | 56933519 | [A/G] |
| 19 | rs11671664 | 5021 | 46172278 | [A/G] |
| 3 | rs11708067 | 5366 | 123065778 | [A/G] |
| 4 | rs11723864 | 1953 | 174574406 | [C/G] |
| 4 | rs11725853 | 2091 | 175642699 | [T/C] |
| 6 | rs11752643 | 2163 | 32669373 | [T/C] |
| 12 | rs1187415 | 5328 | 124491529 | [G/C] |
| 2 | rs11886047 | 1225 | 43850590 | [T/C] |
| 1 | rs12027542 | 4161 | 233340154 | [A/G] |
| 16 | rs12051272 | 3399 | 82663288 | [A/C] |
| 1 | rs12145833 | 2927 | 243483754 | [A/C] |
| 17 | rs12150053 | 2232 | 1664469 | [T/C] |
| 12 | rs12229654 | 813 | 111414461 | [T/G] |
| 10 | rs12249281 | 895 | 5075493 | [T/C] |
| 10 | rs12255372 | 901 | 114808902 | [T/G] |
| 11 | rs12272004 | 1935 | 116603724 | [A/C] |
| 11 | rs12286929 | 154 | 115022404 | [T/C] |
| 15 | rs12437854 | 1862 | 94141833 | [T/G] |
| 2 | rs12463617 | 3031 | 629244 | [A/C] |
| 2 | rs12478601 | 1030 | 43721508 | [A/G] |
| 5 | rs12517906 | 3072 | 180170819 | [T/C] |
| 7 | rs12531570 | 10 | 3109177 | [T/C] |
| 16 | rs12597579 | 3481 | 20257867 | [T/C] |
| 17 | rs12602486 | 2738 | 42241929 | [A/C] |
| 5 | rs12655917 | 2954 | 77291940 | [T/C] |
| 8 | rs12678919 | 4008 | 19844222 | [A/G] |
| 1 | rs12740374 | 4072 | 109817590 | [A/C] |
| 16 | rs12917707 | 101 | 20367690 | [T/G] |
| 17 | rs12943590 | 5073 | 19619998 | [A/G] |
| 3 | rs13081389 | 5074 | 12289800 | [A/G] |
| 7 | rs1319501 | 3938 | 105925753 | [T/C] |
| 8 | rs13266634 | 430 | 118184783 | [A/G] |
| 8 | rs13279522 | 215 | 66974252 | [T/C] |
| 9 | rs1333042 | 3027 | 22103813 | [T/C] |
| 9 | rs1333049 | 2807 | 22125503 | [C/G] |
| 17 | rs13342692 | 1749 | 6946287 | [A/G] |
| 2 | rs13405728 | 6131 | 48978159 | [A/G] |
| 2 | rs13429458 | 2999 | 43638838 | [A/C] |
| 7 | rs1362363 | 915 | 29511689 | [T/C] |
| 2 | rs1367117 | 1311 | 21263900 | [T/C] |
| 3 | rs1374910 | 742 | 185531661 | [T/C] |
| 11 | rs1387153 | 952 | 92673828 | [T/C] |
| 16 | rs1421085 | 2277 | 53800954 | [T/C] |
| 19 | rs1423096 | 1601 | 7739177 | [T/C] |
| 2 | rs1440072 | 2166 | 223936738 | [A/G] |
| 6 | rs145450955 | 1826 | 160671651 | [T/C] |
| 17 | rs146901447 | 5413 | 19616017 | [A/G] |
| 3 | rs1470579 | 756 | 185529080 | [T/G] |
| 3 | rs1501299 | 2180 | 186571123 | [A/C] |
| 15 | rs1532085 | 4125 | 58683366 | [T/C] |
| 16 | rs1532624 | 480 | 57005479 | [A/C] |
| 6 | rs1535435 | 2828 | 135757022 | [T/C] |
| 12 | rs1544410 | 5449 | 48239835 | [A/G] |
| 16 | rs1558902 | 1987 | 53803574 | [A/T] |
| 7 | rs1617640 | 1312 | 100317298 | [T/G] |
| 11 | rs163182 | 1269 | 2844216 | [G/C] |
| 3 | rs1648707 | 3913 | 186551711 | [T/G] |
| 10 | rs16926246 | 4136 | 71093392 | [A/G] |
| 2 | rs16944 | 1079 | 113594867 | [T/C] |
| 11 | rs1695 | 4046 | 67352689 | [A/G] |
| 1 | rs17024258 | 5133 | 110147321 | [T/C] |
| 1 | rs1704198 | 3858 | 213910494 | [T/G] |
| 5 | rs17053082 | 113 | 155394230 | [A/G] |
| 3 | rs17058639 | 3057 | 57882601 | [A/G] |
| 1 | rs17111684 | 2862 | 55625548 | [T/C] |
| 11 | rs1712790 | 5310 | 114621469 | [A/G] |
| 8 | rs17150703 | 3814 | 9745798 | [A/G] |
| 19 | rs17249141 | 4075 | 11200008 | [A/G] |
| 4 | rs17319721 | 1634 | 77368847 | [A/G] |
| 3 | rs17366568 | 1888 | 186570453 | [T/C] |
| 14 | rs1749718 | 2879 | 91183327 | [T/C] |
| 9 | rs17584499 | 5246 | 8879118 | [A/G] |
| 1 | rs1764391 | 5958 | 35260769 | [A/G] |
| 18 | rs17700144 | 1550 | 57811982 | [T/C] |
| 16 | rs17817964 | 5394 | 53828066 | [T/C] |
| 11 | rs1799854 | 5854 | 17448704 | [T/C] |
| 11 | rs1799859 | 1841 | 17419279 | [T/C] |
| 7 | rs1799884 | 5817 | 44229068 | [A/G] |
| 6 | rs1799945 | 2461 | 26091179 | [G/C] |
| 7 | rs1799983 | 5815 | 150696111 | [T/G] |
| 6 | rs1800562 | 5898 | 26093141 | [T/C] |
| 6 | rs1800625 | 4029 | 32152442 | [A/G] |
| 6 | rs1800629 | 4020 | 31543031 | [T/C] |
| 7 | rs1800795 | 1439 | 22766645 | [G/C] |
| 1 | rs1800871 | 3937 | 6946634 | [A/G] |
| 1 | rs1800896 | 4133 | 206946897 | [T/C] |
| 1 | rs1801133 | 629 | 11856378 | [T/C] |
| 10 | rs1801239 | 4099 | 16919052 | [A/G] |
| 2 | rs1801278 | 2322 | 227660544 | [A/G] |
| 3 | rs1801282 | 3625 | 12393125 | [G/C] |
| 18 | rs1805081 | 1046 | 21140432 | [T/C] |
| 2 | rs1861612 | 5933 | 230522398 | [A/G] |
| 19 | rs1862513 | 2208 | 7733793 | [G/C] |
| 3 | rs1866813 | 4012 | 136801938 | [T/G] |
| 6 | rs1867351 | 1825 | 160543123 | [A/G] |
| 11 | rs1894116 | 3962 | 102070639 | [A/G] |
| 14 | rs1957894 | 1151 | 61908111 | [A/C] |
| 1 | rs1993709 | 984 | 72838529 | [T/C] |
| 6 | rs2010963 | 3562 | 43738350 | [C/G] |
| 8 | rs2073617 | 6123 | 119964283 | [T/C] |
| 8 | rs2073618 | 2808 | 119964052 | [G/C] |
| 12 | rs2074356 | 300 | 112645401 | [T/C] |
| 22 | rs2106294 | 619 | 31645759 | [T/C] |
| 10 | rs2116830 | 1063 | 78646536 | [A/C] |
| 20 | rs2179357 | 209 | 45371084 | [A/G] |
| 6 | rs2207396 | 1002 | 152382382 | [T/C] |
| 6 | rs2227956 | 56 | 31778272 | [A/G] |
| 1 | rs2229238 | 5091 | 154437896 | [T/C] |
| 14 | rs2230087 | 1241 | 23495243 | [T/C] |
| 20 | rs2235491 | 3934 | 45354291 | [A/G] |
| 11 | rs2237892 | 4283 | 2839751 | [A/G] |
| 11 | rs2237895 | 5350 | 2857194 | [A/C] |
| 11 | rs2237897 | 4950 | 2858546 | [T/C] |
| 11 | rs2266788 | 2312 | 116660686 | [T/C] |
| 12 | rs2272046 | 1989 | 66224461 | [A/C] |
| 19 | rs2278426 | 1309 | 11350488 | [A/G] |
| 6 | rs2282143 | 1682 | 160557643 | [A/G] |
| 11 | rs2283228 | 1111 | 2849530 | [A/C] |
| 17 | rs2289669 | 1037 | 19463343 | [T/C] |
| 11 | rs2334499 | 5285 | 1696849 | [T/C] |
| 12 | rs2358944 | 1893 | 66117558 | [T/C] |
| 9 | rs2383208 | 6140 | 22132076 | [A/G] |
| 8 | rs2410601 | 2249 | 18922577 | [G/C] |
| 20 | rs2425904 | 2204 | 45356876 | [A/G] |
| 20 | rs2425911 | 3919 | 45360219 | [C/G] |
| 15 | rs2467853 | 515 | 45698793 | [T/G] |
| 16 | rs247617 | 5968 | 56990716 | [A/C] |
| 9 | rs2479106 | 682 | 126525212 | [A/G] |
| 5 | rs247916 | 1382 | 87530253 | [A/C] |
| 1 | rs2483058 | 4004 | 206626828 | [G/C] |
| 5 | rs2591797 | 2442 | 62078070 | [T/C] |
| 1 | rs2605100 | 5157 | 219644224 | [A/G] |
| 5 | rs261967 | 3014 | 95850250 | [A/C] |
| 8 | rs2648875 | 1272 | 129072161 | [A/G] |
| 3 | rs266717 | 4044 | 186530484 | [T/C] |
| 3 | rs266719 | 5297 | 186501648 | [T/C] |
| 3 | rs266729 | 1647 | 186559474 | [C/G] |
| 8 | rs268 | 1838 | 19813529 | [T/C] |
| 2 | rs2681019 | 1333 | 23187504 | [A/C] |
| 13 | rs2765086 | 1938 | 24504955 | [A/G] |
| 2 | rs2867125 | 5035 | 622827 | [A/G] |
| 10 | rs290487 | 5076 | 114909731 | [T/C] |
| 2 | rs2943650 | 2733 | 227105921 | [T/C] |
| 20 | rs3091904 | 5846 | 45361455 | [A/G] |
| 20 | rs3092412 | 3896 | 45350096 | [T/A] |
| 1 | rs3101336 | 3789 | 72751185 | [A/G] |
| 6 | rs316019 | 5930 | 160670282 | [T/G] |
| 7 | rs3211938 | 5342 | 80300449 | [A/C] |
| 6 | rs34059508 | 747 | 160575837 | [T/C] |
| 6 | rs34130495 | 1876 | 160560824 | [T/C] |
| 17 | rs35395280 | 634 | 19480643 | [T/G] |
| 17 | rs35790011 | 5003 | 19463591 | [A/G] |
| 19 | rs3745367 | 153 | 7734511 | [T/C] |
| 21 | rs3746876 | 2059 | 39671447 | [A/G] |
| 16 | rs3751812 | 5985 | 53818460 | [T/G] |
| 4 | rs3755863 | 1753 | 23815522 | [A/G] |
| 15 | rs3759890 | 2361 | 45314594 | [G/C] |
| 2 | rs3791783 | 4071 | 190924163 | [T/C] |
| 2 | rs3792269 | 1689 | 241531479 | [A/G] |
| 6 | rs3798220 | 5800 | 160961137 | [A/G] |
| 9 | rs3802457 | 2402 | 97741336 | [A/G] |
| 16 | rs3865188 | 4908 | 82650717 | [T/A] |
| 3 | rs3912607 | 265 | 162498758 | [A/G] |
| 17 | rs391300 | 178 | 2216258 | [A/G] |
| 12 | rs4149056 | 2960 | 21331549 | [A/G] |
| 10 | rs4244285 | 4116 | 96541616 | [A/G] |
| 19 | rs429358 | 2062 | 45411941 | [A/G] |
| 5 | rs4311394 | 764 | 53300662 | [A/G] |
| 2 | rs4383389 | 4951 | 133208597 | [T/C] |
| 3 | rs4402960 | 1882 | 185511687 | [A/C] |
| 2 | rs4417767 | 3972 | 133205288 | [A/C] |
| 19 | rs4420638 | 656 | 45422946 | [T/C] |
| 9 | rs4446815 | 1145 | 22847961 | [A/G] |
| 19 | rs445925 | 950 | 45415640 | [A/G] |
| 10 | rs4506565 | 2913 | 114756041 | [T/A] |
| 6 | rs461473 | 1559 | 160543562 | [T/C] |
| 6 | rs4646272 | 1688 | 160551093 | [A/C] |
| 17 | rs46522 | 186 | 46988597 | [T/C] |
| 16 | rs4673 | 3606 | 88713236 | [T/C] |
| 6 | rs4712523 | 2360 | 20657564 | [A/G] |
| 19 | rs472265 | 5348 | 39580737 | [A/G] |
| 12 | rs4767631 | 814 | 118311743 | [T/C] |
| 18 | rs476828 | 4002 | 57852587 | [A/G] |
| 15 | rs4776970 | 6107 | 68080886 | [A/T] |
| 16 | rs4780476 | 1693 | 12862007 | [A/C] |
| 16 | rs4783244 | 67 | 82662268 | [A/C] |
| 18 | rs4796955 | 5176 | 12262402 | [A/C] |
| 6 | rs4880 | 2833 | 160113872 | [A/G] |
| 2 | rs4971516 | 3470 | 20903015 | [A/G] |
| 2 | rs4972593 | 2406 | 174462854 | [T/A] |
| 9 | rs4977574 | 1326 | 22098574 | [T/C] |
| 10 | rs4986893 | 3440 | 96540410 | [A/G] |
| 10 | rs501120 | 920 | 44753867 | [A/G] |
| X | rs5030868 | 1644 | 153762634 | [A/G] |
| X | rs5031002 | 331 | 66942625 | [T/C] |
| 2 | rs515135 | 5257 | 21286057 | [A/G] |
| 11 | rs5210 | 3049 | 17408251 | [A/G] |
| 11 | rs5219 | 1628 | 17409572 | [T/C] |
| 13 | rs534870 | 2398 | 80959207 | [A/G] |
| 1 | rs543874 | 3160 | 177889480 | [A/G] |
| 8 | rs545854 | 6062 | 9860080 | [G/C] |
| 19 | rs5498 | 5894 | 10395683 | [A/G] |
| 11 | rs564343 | 2179 | 65895166 | [T/C] |
| 18 | rs571312 | 2995 | 57839769 | [A/C] |
| 2 | rs5742904 | 720 | 21229160 | [T/C] |
| 1 | rs5744168 | 859 | 223285200 | [A/G] |
| 12 | rs5888 | 1331 | 124800202 | [T/C] |
| X | rs5945326 | 3987 | 152899922 | [T/C] |
| 1 | rs599839 | 1671 | 109822166 | [A/G] |
| 20 | rs6066059 | 5057 | 45368154 | [T/C] |
| 16 | rs62033400 | 5799 | 53811788 | [A/G] |
| 6 | rs622342 | 2420 | 160572866 | [A/C] |
| 5 | rs6232 | 4057 | 95751785 | [A/G] |
| 5 | rs6235 | 2501 | 95728898 | [C/G] |
| 17 | rs6257 | 2369 | 7533717 | [A/G] |
| 17 | rs6259 | 6030 | 7536527 | [T/C] |
| 1 | rs629301 | 2152 | 109818306 | [A/C] |
| 1 | rs646776 | 4092 | 109818530 | [A/G] |
| 19 | rs6511720 | 969 | 11202306 | [T/G] |
| 11 | rs652722 | 4086 | 31905534 | [A/G] |
| 2 | rs6545814 | 3734 | 25131316 | [T/C] |
| 18 | rs6567160 | 33 | 57829135 | [A/G] |
| 15 | rs6576507 | 3804 | 26288394 | [T/C] |
| 11 | rs6589566 | 3981 | 116652423 | [T/C] |
| 11 | rs659366 | 1267 | 73694754 | [T/C] |
| 11 | rs660339 | 2753 | 73689104 | [A/G] |
| 7 | rs662 | 3901 | 94937446 | [T/C] |
| 6 | rs662301 | 3498 | 160696919 | [A/G] |
| 11 | rs662799 | 1078 | 116663707 | [T/C] |
| 1 | rs6684514 | 102 | 156255456 | [A/G] |
| 12 | rs671 | 6130 | 112241766 | [A/G] |
| 2 | rs6712932 | 5858 | 105837598 | [T/C] |
| 2 | rs6723108 | 5118 | 135479980 | [T/G] |
| 2 | rs6746030 | 1772 | 167099158 | [A/G] |
| 2 | rs6756629 | 6016 | 44065090 | [A/G] |
| 3 | rs6773957 | 2205 | 186573705 | [A/G] |
| 6 | rs683369 | 3979 | 160551204 | [G/C] |
| 4 | rs6857327 | 2651 | 40954272 | [A/G] |
| 6 | rs6921953 | 913 | 16019115 | [T/C] |
| 6 | rs6930576 | 5293 | 148704954 | [T/C] |
| 1 | rs699 | 607 | 230845794 | [A/G] |
| 6 | rs699947 | 651 | 43736389 | [A/C] |
| 8 | rs7002832 | 1563 | 32054373 | [A/G] |
| 9 | rs7018475 | 2298 | 2137685 | [A/C] |
| 9 | rs7020996 | 1777 | 22129579 | [T/C] |
| 7 | rs705381 | 4974 | 94953949 | [A/G] |
| 12 | rs705702 | 6117 | 56390636 | [A/G] |
| 16 | rs708272 | 1006 | 56996288 | [A/G] |
| 15 | rs7119 | 4305 | 77777632 | [A/G] |
| 16 | rs7190492 | 3857 | 53828752 | [A/G] |
| 18 | rs7234864 | 1595 | 57734857 | [A/G] |
| 20 | rs734784 | 3968 | 43723627 | [T/C] |
| 19 | rs7412 | 1276 | 45412079 | [T/C] |
| 10 | rs7474896 | 4022 | 37982097 | [T/C] |
| 7 | rs7493 | 3495 | 95034775 | [C/G] |
| 17 | rs75493593 | 4041 | 6945087 | [A/C] |
| 11 | rs757110 | 2342 | 17418477 | [T/G] |
| 2 | rs7581414 | 2789 | 133231758 | [T/G] |
| 2 | rs7583877 | 1319 | 100460654 | [A/G] |
| 2 | rs7588550 | 1790 | 213168768 | [A/G] |
| 2 | rs7607980 | 130 | 165551201 | [A/G] |
| 7 | rs7636 | 4069 | 100490077 | [T/C] |
| 17 | rs76645859 | 2308 | 19476126 | [T/C] |
| 5 | rs7703051 | 2729 | 74625487 | [A/C] |
| 5 | rs7725 | 2257 | 179727957 | [T/C] |
| 9 | rs773506 | 1000 | 93975471 | [A/G] |
| 17 | rs77474263 | 2932 | 19451364 | [T/C] |
| 6 | rs7754840 | 2994 | 20661250 | [C/G] |
| 17 | rs77630697 | 1850 | 19445761 | [T/C] |
| 6 | rs7766070 | 2328 | 20686573 | [T/G] |
| 6 | rs7769051 | 2320 | 133146796 | [A/C] |
| 10 | rs7903146 | 2057 | 114758349 | [A/G] |
| 10 | rs7919006 | 1784 | 76808760 | [T/C] |
| 10 | rs7923837 | 2962 | 94481917 | [A/G] |
| 14 | rs8014194 | 51 | 95720678 | [T/A] |
| 16 | rs8044769 | 5906 | 53839135 | [T/C] |
| 16 | rs8050136 | 2776 | 53816275 | [A/C] |
| 17 | rs8065082 | 233 | 19465191 | [A/G] |
| 19 | rs8111699 | 3826 | 1209714 | [G/C] |
| 2 | rs815815 | 2906 | 47399064 | [A/G] |
| 6 | rs8177508 | 2043 | 160677671 | [T/C] |
| 6 | rs8177516 | 1776 | 160664685 | [A/G] |
| 11 | rs8181588 | 4061 | 2831541 | [T/C] |
| 6 | rs8187717 | 5058 | 160769797 | [A/C] |
| 6 | rs8187725 | 5290 | 160858154 | [A/G] |
| 4 | rs8192678 | 5177 | 23815662 | [T/C] |
| 7 | rs854560 | 3468 | 94946084 | [T/A] |
| 7 | rs854572 | 2861 | 94954696 | [C/G] |
| 3 | rs864265 | 5381 | 186554292 | [A/C] |
| 14 | rs878889 | 5184 | 57221139 | [A/G] |
| 6 | rs911946 | 2358 | 169043500 | [T/C] |
| 20 | rs926392 | 1335 | 37690464 | [A/G] |
| 9 | rs9282541 | 1340 | 107620835 | [A/G] |
| 11 | rs9300039 | 5127 | 41915366 | [A/C] |
| 21 | rs9305354 | 1060 | 29475196 | [T/C] |
| 6 | rs9348440 | 1740 | 20641336 | [A/G] |
| 6 | rs9349379 | 2831 | 12903957 | [A/G] |
| 6 | rs9356744 | 5874 | 20685486 | [A/G] |
| 2 | rs939583 | 3453 | 622531 | [T/C] |
| 14 | rs942740 | 398 | 91158869 | [T/C] |
| 9 | rs944797 | 2016 | 22115286 | [A/G] |
| 6 | rs9465871 | 5104 | 20717255 | [T/C] |
| 6 | rs9494266 | 3038 | 135851573 | [A/G] |
| 13 | rs9552911 | 3906 | 23864657 | [T/C] |
| 11 | rs964184 | 5829 | 116648917 | [C/G] |
| 7 | rs9770242 | 674 | 105926331 | [A/C] |
| 3 | rs9813516 | 989 | 60293004 | [T/C] |
| 1 | rs984222 | 5784 | 119503843 | [C/G] |
| 6 | rs987237 | 1711 | 50803050 | [T/C] |
| 11 | rs988712 | 2297 | 27563382 | [T/G] |
| 16 | rs9930333 | 1955 | 53799977 | [T/G] |
| 16 | rs9930506 | 4997 | 53830465 | [A/G] |
| 16 | rs9939609 | 2966 | 53820527 | [A/T] |
| 16 | rs9940128 | 3461 | 53800754 | [T/C] |
| 16 | rs9941349 | 3500 | 53825488 | [T/C] |
| 16 | rs9989419 | 5237 | 56985139 | [T/C] |
