# Supplementary material for: Genomic variants associated with type 2 diabetes mellitus among Filipinos
Source: PLoS One. 2024 Nov 19;19(11):e0312291. doi: 10.1371/journal.pone.0312291 (PMC11575783; doi:10.1371/journal.pone.0312291)
Supplement: S1 File — (DOCX) [file pone.0312291.s005.docx]

**Supplementary Material 1.** Inclusion and exclusion criteria for enrollment of participants in the study

The general inclusion criteria were the following: Filipinos above the age of 18, unrelated, and able to provide informed consent independently.

For the T2DM group, participants satisfied the general criteria and any of the criteria for T2DM as defined by the American Diabetes Association (ADA, 2018), which include the presence of any of the following: (1) fasting blood sugar (FBS) ≥ 126 mg/dL (7.0 mmol/L), with fasting defined as without caloric intake for at least 8 hours; (2) 2-hour plasma glucose ≥ 200 mg/dL (11.1 mmol/L) during oral glucose tolerance test (2-hr OGTT), which is performed using a glucose load containing the equivalent of 75g anhydrous glucose dissolved in water; (3) glycosylated hemoglobin A1C (HbA1C) ≥ 6.5% (48 mmol/L), which is performed in a laboratory using methods certified by the National Glycohemoglobin Standardization Program (NGSP), standardized to the Diabetes Control and Complications Trial (DCCT) assay; or (4) a random plasma glucose ≥ 200 mg/dL (11.1 mmol/L) in the presence of classic symptoms of hyperglycemia or hyperglycemic crisis.

For non-diabetic controls, participants satisfied the general criteria and none of the criteria for T2DM group. Also, they should have no family history of diabetes mellitus among first-degree relatives.

The following were excluded from the study: (1) previously diagnosed with Type 1 diabetes mellitus; (2) currently pregnant or lactating; (3) with active alcohol abuse or illicit drug use within the past three months; (4) with malignancy with active systemic disease; or (5) diagnosed with malignancy and disease-free for less than five (5) years.
